# Clinical and Molecular Characterization of a Novel Homozygous Frameshift Variant in AEBP1-Related Classical-like Ehlers Danlos Syndrome Type 2 with Comparison to Previously Reported Rare Cases

**DOI:** 10.3390/genes15040461

**Published:** 2024-04-06

**Authors:** Zong Yi Ha, Chieko Chijiwa, Suzanne Lewis

**Affiliations:** 1 Department of Medical Genetics, University of British Columbia, C234-4500 Oak Street, Vancouver, BC V6H 3N1, Canadasuzanne.lewis@ubc.ca (S.L.); 2 The BC Provincial Medical Genetics Program, University of British Columbia, C234-4500 Oak Street, Vancouver, BC V6H 3N1, Canada; 3 BC Children’s Hospital Research Institute, University of British Columbia, C234-4500 Oak Street, Vancouver, BC V6H 3N1, Canada

**Keywords:** Ehlers-Danlos syndrome, classical-like EDS type 2 (clEDS2), adipocyte enhancer binding protein 1 (AEBP1), aortic carboxypeptidase-like protein (ACLP), autosomal recessive, connective tissue disorders, short stature, prematurity

## Abstract

Recently, an autosomal recessive subtype of connective tissue disorder within the spectrum of Ehlers–Danlos syndrome (EDS), named classical-like EDS type 2 (clEDS2), was identified. clEDS2 is associated with biallelic variants in the adipocyte enhancer binding protein 1 (*AEBP1*) gene, specifically, affecting its aortic carboxypeptidase-like protein (ACLP) isoform. We described the 15th patient (13th family) diagnosed with clEDS2. This patient presented with notable similarities in phenotype to the documented cases, along with additional characteristics such as significant prematurity and short stature. An EDS sequencing panel-based analysis revealed homozygous AEBP1: NM_001129.5:c.2923del, p.Ala975Profs*22 likely pathogenic variants, and maternally inherited heterozygous COL11A1: NM_001854.4:c.1160A>G, p.Lys387Arg variant of uncertain significance in our patient. Upon comprehensive review of all previously reported clEDS2 patients, our patient exhibited the following overlapping phenotypes, including cutaneous features: hyperextensibility, atrophic scars/delayed wound healing (100%), easy bruising (100%), excessive skin (93%); skeletal features: generalized joint hypermobility (93%), pes planus (93%), dislocation/subluxation (93%); and cardiovascular features (86%). Our patient did not display symptoms of the critical complications reported in a few individuals, including superior mesenteric artery aneurysms and ruptures, aortic root aneurysm/dissection, spontaneous pneumothoraxes, and bowel ruptures. Together, this case expands the genetic and clinical phenotypic spectrum of AEBP1-related clEDS2.

## 1. Introduction

Ehlers-Danlos syndrome (EDS), with an estimated prevalence of 1/5000, comprises a group of clinically heterogeneous heritable connective tissue disorders characterized by skin hyperextensibility, joint hypermobility, and connective tissue fragility. The 2017 International Classification recognizes 13 distinct subtypes of EDS and diverse pathogenic variants in 19 genes that mainly encode fibrillar collagens, collagen-modifying proteins, or extracellular matrix synthesis enzymes [1]. Classical-like EDS (clEDS) is a rare type of autosomal recessive EDS that is further categorized based on genetic causes: classical-like EDS type 1 (clEDS1) MIM #606408 is associated with variants in the *TNXB* gene [1], and classical-like EDS type 2 (clEDS2) MIM #618000 is associated with variants in the *AEBP1* gene [2,3]. Although there are many overlapping clinical features between clEDS1 and clEDS2, clEDS2 is reported to have additional features of atrophic scarring, early onset osteopenia, and cardiovascular features [2,4].

*AEBP1* encodes two protein isoforms: adipocyte enhancer binding protein 1 (AEBP1), which is a transcription repressor that negatively regulates adipogenesis and smooth muscle cell differentiation [5] and aortic carboxypeptidase-like protein (ACLP), which is an extracellular matrix (ECM) protein identified in the dermis, periosteum, blood vessels, and lung basement membrane with fundamental roles in embryogenesis and ECM repair and maintenance [6,7]. clEDS2 is specifically linked to the expression, localization, and function of the ACLP protein [6]. To date, 14 patients from 12 families with clEDS2 have been described [3,4,6,8,9,10,11,12].

In this study, we identify a novel homozygous pathogenic variant in AEBP1 in a 16-year-old female patient of Syrian descent with a clinical diagnosis of clEDS2, short stature, and premature birth, and compare our patient phenotypically and genetically to previously reported *AEBP1*-related clEDS2 patients from 12 families.

## 2. Materials and Methods

The following study was conducted in cooperation with the British Columbia Provincial Medical Genetics Program. The patient and the parents of the patient were recruited for genetic testing. Informed consent for the presentation of clinical data was obtained from all subjects involved in the study.

## 3. Results

### 3.1. Case Report

Our patient is one of eight children of non-consanguineous, healthy parents. Both parents are of Syrian descent, but they come from different regions. No skin hyperextensibility, atopic scarring, or joint hypermobility were noted in her parents or her siblings.

She was healthy upon premature delivery at 32 weeks of gestation. However, she required monitoring in the hospital for feeding and apnea until she was discharged home after 15 days. She had a bilateral congenital hip dislocation, which was surgically corrected. At the age of 12 years, multiple varicose veins developed in her right lower leg with focal pain and muscle weakness. In her teenage years, she experienced frequent muscle fatigue and sought medical attention. She had a history of very stretchy, easy-bruising skin, and hypermobile joints. Her other concerns were multifocal joint pain, small stature, and dry, brittle hair. She reported eating a very limited quantity of food at meals to avoid the uncomfortable feeling of heaviness in her stomach. There was no history of dislocations or fractures. There was also no history of myopia, hearing issues, dental concerns, herniation, fainting, tachycardia, nausea, food intolerance, or bladder concerns. Furthermore, there were no concerns regarding developmental delay or intellectual disability.

At the age of 16 years, our patient’s height measured 140 cm (−3.34 standard deviations), her weight measured 35 kg (−3.26 standard deviations), and her occipital frontal circumference measured 50.5 cm (−2.69 standard deviations).

On physical assessment, she exhibited a unique facial appearance characterized by an exceptionally narrow and thin face, a slender nasal root, with a normal appearance of her eyes and ears in terms of shape, size, and positioning. Her shoulder-length hair was notably sparse, dry, and brittle. Her skin was described as exceedingly thin and demonstrated an unusual degree of hyperextensibility, a feature particularly pronounced around her elbow area. This hyperextensibility was coupled with a degree of translucency. Furthermore, her skin bore multiple ‘tissue-paper’ scars across her arms, knees, and shins, alongside a large keloid scar on her knee, evidencing a tendency for atypical wound healing and scar formation.

Musculoskeletal evaluation revealed widespread joint hypermobility, achieving a Beighton score of 9 out of 9. This was accompanied by notable finger joint hyperlaxity and the presence of bilateral pes planus. Besides her joint issues, she had multiple varicose veins localized to her right calf, with associated pain and muscle weakness. She had no other vascular findings.

The neurological examination was grossly normal. Cranial nerve evaluation was normal, demonstrating full extraocular eye movements as well as full facial muscle strength and sensation in all branches of the facial nerves. Tongue and palate evaluations were normal, including the midline uvula, and there were no signs of fasciculations. Neck extension and flexion were normal. Motor function was also deemed normal, with appropriate muscle tone and strength and no signs of upper motor neuron dysfunction such as the Babinski sign or clonus. Reflexes were normal, graded 2+ in all muscle groups of the upper and lower limbs. The cardiorespiratory, genitourinary, and gastrointestinal systems were evaluated and found to be within normal limits.

Doppler ultrasound on bilateral lower extremities confirmed superficial varicose veins as well as excluded deep venous thrombosis and other vascular malformations. A full spine X-ray revealed lateral disc herniation between L5 and S1. A skeletal survey showed translucency along her sagittal suture with additional indistinct translucency in the occiput on lateral projection, yet no aggressive osseous abnormality was present. Nerve conduction studies and electromyography were normal and ruled out underlying peripheral neuropathy, compressive mononeuropathy, or myopathy. An urgent echocardiogram referral was sent, but it had not been conducted. Considering the possibility of overlapping symptoms with Stickler syndrome, we referred the patient for formal assessments in audiology and ophthalmology to ensure a comprehensive evaluation and appropriate management of her condition. Computed tomography was also considered for ruling out aneurysms.

### 3.2. Molecular Analysis

**Figure 1:**
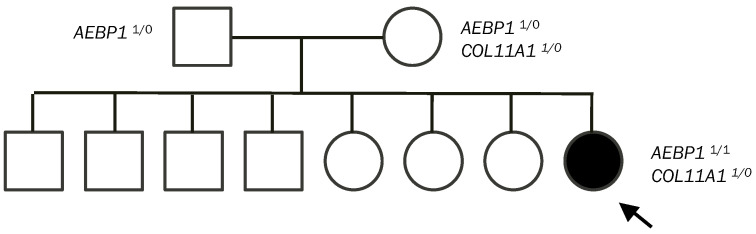
Pedigree of the familial genetic segregation of the *AEBP1*^1/0^ and *COL11A*^1/0^ variants in our patient and her parents. Arrow points to our patient. *AEBP1*^1/0^ is the heterozygous c.2923del variant. *AEBP1*^1/1^ is the homozygous c.2923del variant. *COL11A*^1/0^ is a heterozygous c.1160A>G variant.

Blueprint Genetics Ehlers-Danlos Syndrome Panel (41 genes) [13] identified three variants in our patient: two identical homozygous variants in *AEBP1* c.2923del and a heterozygous variant in *COL11A1* c.1160A>G. Parental testing of these variants revealed both asymptomatic parents were heterozygous for the *AEBP1* c.2923del variant. The *COL11A1* c.1160A>G variant was identified as being maternally inherited (Figure 1). Since the siblings of our patient did not have any similar clinical symptoms, they were not tested.

The *AEBP1* c.2923del variant is predicted to result in a frameshift in exon 19, which is predicted to introduce a premature termination codon p.Ala975Profs*22. This truncates the ACLP protein by 163 AA and reduces the peptide length by over 10 percent. This variant is classified as a likely pathogenic variant, according to the American College of Medical Genetics and Genomics (ACMG). It is absent in large reference population databases: gnomAD, ClinVar, and HGMD [14,15,16].

The *COL11A1* c.1160A>G variant is classified as a variant of uncertain significance (VUS) by ACMG. It is predicted to introduce a missense codon, p.Lys387Arg, which lays in the non-conserved region between the thrombospondin N-terminal-like domain and the first Glycine-X-Y triple helices region [17]. It is also absent in large reference population databases: gnomAD, ClinVar, and HGMD [14,15,16]. Pathogenic monoallelic variants in the *COL11A1* gene are primarily linked to autosomal dominant Stickler syndrome type II (STL2) MIM #604841, Marshall syndrome (MRSHS) MIM #154780, and non-syndromic hearing loss (ADNSHL) at the DFNA37 locus MIM #618533 [17]. Our patient and her mother do not exhibit any key phenotypic characteristics of STL2 or MRSHS syndromes, including myopia, astigmatism, vitreous anomalies, or sensorineural hearing loss [17]. Thus, this *COL11A1* c.1160A>G variant is likely not significant to our patient’s phenotype.

## 4. Discussion

We have identified a 15th clEDS2 patient (13th family) with a novel homozygous pathogenic variant in *AEBP1*. Comprehensive clinical and molecular comparisons of our patient to all previously reported individuals with clEDS2 are recorded in Table S1.

clEDS2 appears to be rare, with only 14 previously reported individuals described in the literature [3,4,6,8,9,10,11,12]. Including our patient, this syndrome is reported in nine females and six males, and the median age at the time of diagnosis was 35 years with a range of 12–65 years old (Table S1).

The features shared between most of the individuals with biallelic pathogenic variants in *AEBP1* are cutaneous features: hyperextensibility (100%), atrophic scars/delayed wound healing (100%), easy bruising (100%), excessive skin (93%); skeletal features: generalized joint hypermobility (93%), pes planus (93%), dislocation/subluxation (93%); and cardiovascular features (86%) (Table S1). Critical complications are rare, occurring in one or two patients, and include multiple superior mesenteric artery aneurysms and ruptures, aortic root aneurysms or dissections requiring surgery, spontaneous pneumothorax, and bowel ruptures requiring anastomosis of the bowel and colostomy [4,6,11]. The congenital presentation of these individuals can include hypotonia, gross and fine motor developmental delay, and congenital hip dislocations (Table S1). clEDS2 patients can have wide atrophic scars in various parts of the body, which may present with a cigarette paper, hemosiderotic, or papyraceous appearance (Table S1). Other reported cardiovascular features include aortic root dilation, carotid stenosis, mitral valve regurgitation/prolapse, discovered at ages 33, 35, 39, and 58 years, varicose veins, dissection, dilation, and tortuosity of arteries, discovered at ages 53 and 63 years, and hematoma [3,6,10,12]. Features reported in a few patients include dental abnormalities (60%), hernias (60%), alopecia (53%), scoliosis/kyphoscoliosis (53%), neuromuscular features (53%), and osteopenia (47%). Fifty percent (3/6) of male patients presented with cryptorchidism (Table S1). Three patients presented with multiple subtle signs of autonomic disfunction, such as urinary retention, bowel immobilization, postural orthostatic tachycardia syndrome, and chronic fatigue (Table S1). Two other patients presented with only chronic fatigue (Table S1). Interestingly, 47 percent of all patients were born prematurely, between 30 and 36 weeks of gestation (Table S1). Our patient and one other individual with premature birth at 32 and 30 weeks of gestation, respectively, were reported to have significant symmetric short stature at 140 cm (−3.34 standard deviations) and 150 cm (−2.01 standard deviations) and measured significantly below their mid-parental heights (Table S1) [3]. Furthermore, while two patients gave birth to healthy children, both had obstetric complications of significant postpartum bleeding [10,11]. One of the two individuals had severe paralytic ileus post-caesarean section and postoperative abdominal hernia [11].

Previously reported transmission electron microscopy analysis on the skin biopsies of the individuals with *AEBP1* variants showed abundant collagen flower deposits in the dermis, which are collagen deposits with a ruffled appearance on the edges [3,4,6,8,11]. Elastin deposits ranged from normal to decreased [3,4,6,8,11]. The biochemical analysis showed reduced secretion of type I and type III procollagen [4].

**Figure 2:**
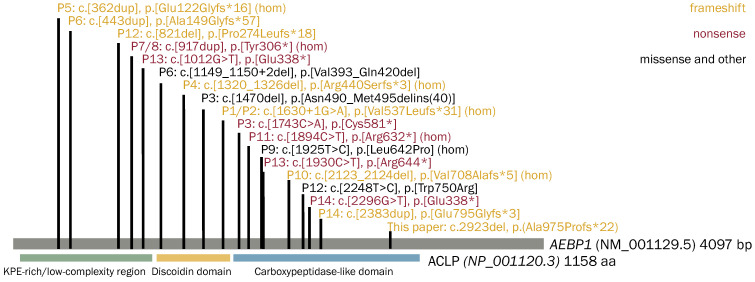
The distribution of potentially pathogenic variants in 15 patients from 13 families with clEDS2 on the illustration of the *AEBP1* gene [3,4,6,8,9,10,11,12]. Domains of the ACLP are shown at the corresponding location. hom: homozygote; P1–P14: Patients 1–14; KPE-rich: lysine, proline, and glutamic acid-rich. Orange represents frameshift variants; red represents nonsense variants; black represents other variants.

A total of 14 out of 18 unique *AEBP1* variants were nonsense, frameshift, and splice site variants, predicted or experimentally confirmed to lead to nonsense-mediated mRNA decay (NMD) (Figure 2). In Patient 3, a 1-bp deletion, c.1470del, in exon 12 led to the retention of intron 12 [6], which resulted in the peptide product being retained intracellularly and not secreted [7]. In Patient 6, c.1149_1150+2del in one allele led to the loss of the last 4 bp of exon 9 and skipping of exon 10, resulting in an in-frame deletion of p.Val383_Gln420del and the mRNA expression was significantly decreased, indicating that the AEBP1 transcript was unstable and/or prone to NMD. In Patient 9, the c.1925T>C p.Leu642Pro variant is predicted in silico to affect the tertiary structure of the protein by disrupting an α-helix located in a highly conserved domain, thus likely interfering with its function in terms of impaired partner binding capability [3]. In Patient 12, the c.2248T>C, p.Trp750Arg variant affects exon 18; however, the underlying mechanism remains unclear [8]. These findings suggest clEDS2 is associated with loss of function, reduced levels, or the absence of ACLP proteins.

In our patient, the p.Ala975Profs*22 variant is downstream of all previously reported pathogenic AEBP1 variants and is predicted to not undergo NMD. Since N1030 is predicted to be absent in this variant and it is a key player in the secretion and cellular function of ACLP [7], the resulting ACLP peptide of the p.Ala975Profs*22 variant may be retained in the cell, thus limiting its downstream functions.

Upon a detailed review of clEDS2, we advocate for a multidisciplinary team approach to effectively manage patients with this condition. This includes patient education, lifestyle modifications, pain management, and screening for critical complications. Lifestyle modifications should entail avoiding activities that may predispose individuals to vascular complications and promoting low-impact sports to maintain physical well-being. Prioritizing pain management is crucial for enhancing the patient’s quality of life. To monitor for potential complications, regular check-ups focusing on the cardiac, vascular, and gastrointestinal systems are essential. An urgent echocardiogram is imperative for detecting aortic dilation or dissection and various valvular defects. Imaging, such as a CT angiogram or MRI, should be considered to identify any asymptomatic arterial aneurysms or dissections. Given the risk of organ rupture, close attention should be paid to gastrointestinal symptoms. Clinical monitoring of subtle autonomic imbalances such as postural orthostatic tachycardia syndrome, chronic fatigue, bowel immobility or obstruction, and urinary retention is also advised. Furthermore, it is important to discuss the increased risks associated with pregnancy in women with clEDS2, emphasizing the necessity for careful monitoring and management. Genetic counseling should be provided regarding the autosomal recessive inheritance within the family.

## 5. Conclusions

In conclusion, this case report expands the current knowledge on the phenotypic spectrum and genotypic variants in the *AEBP1* gene. Additional clinical investigations and molecular analyses are imperative to elucidate the pathophysiology of this disorder.

## Data Availability

Raw data are available upon request to the researchers. The data are not publicly available due to privacy restrictions.

## Supplementary Materials

The following supporting information can be downloaded at: https://www.mdpi.com/article/10.3390/genes15040461/s1, Table S1: Comprehensive clinical and molecular features reported in individuals with clEDS2.
