# Supplementary material for: Clinical and Molecular Characterization of a Novel Homozygous Frameshift Variant in AEBP1-Related Classical-like Ehlers Danlos Syndrome Type 2 with Comparison to Previously Reported Rare Cases
Source: Genes (Basel). 2024 Apr 6;15(4):461. doi: 10.3390/genes15040461 (PMC11049394; doi:10.3390/genes15040461)
Supplement: Supplementary file 1 [file genes-15-00461-s001.zip › genes-2928487-supplementary.pdf]

Supplementary Information

Table S1. Comprehensive clinical and molecular features reported in individuals with clEDS2.

Family No.	I		II	III	IV	V	VI		VII	VIII	IX	X	XI	XII	XIII
Patient No.	1	2	3	4	5	6	7	8	9	10	11	12	13	14	15
Citations	Alazami et al. (2016) Maddirevula et al. (2020)		Blackburn et al. (2018) Vishwanath et al. (2020)		Syx et al. (2019)		Hebebrand et al. (2019)		Ritelli et al. (2019)	Di Giosaffatte et al. (2022)	Sanai et al. (2022)	Angwin (2023)		Yamaguchi et al. (2023)	This Paper
Age (at the reported time)	12	24	35	33	58	21	39	38	53	26	35	62	41	45	16
Sex	Female	Male	Male	Male	Male	Female	Female	Male	Female	Male	Female	Female	Female	Female	Female
Ethnicity	Middle Eastern	Middle Eastern	Cauc-asian	Caucasian	Caucasian	Caucasian	Greek	Greek	Italian	NA	Japanese	Cauc-asian	Cauc-asian	Japanese	Syrian
AEBP1 variant (NM_001129.5)	c.[1630 + 1G>A]; [1630 + 1G>A]	c.[1630 + 1G>A]; [1630 + 1G>A]	c.[1470del]; [1743C>A]	c.[1320_1326del]; [1320_1326del]	c.[362dup]; [362dup]	c.[443dup]; [1149_1150+2del]	c.[917dup]; [917dup]	c.[917dup]; [917dup]	c.[1925T>C]; [1925T>C]	c.[2123_2124del]; [2123_2124del]	c.[1894C>T]; [1894C>T]	c.[821del]; [2248T>C]	c.[1012G>T]; [1930C>T]	c.[2296G>T]; [2383dup]	c.[2923del]; [2923del]
Protein alteration (NP_001120.3)	p.[Val537Leufs*31]; [Val537Leufs*31]	p.[Val537Leufs*31]; [Val537Leufs*31]	p.[Asn490_Met495delins(40)]; [Cys581*]	p.[Arg440Serfs*3]; [Arg440Serfs*3]	p.[Glu122Glyfs*16]; [Glu122Glyfs*16]	p.[Ala149Glyfs*57]; [Val383_Gln420del]	p.[Tyr306*]; [Tyr306*]	p.[Tyr306*]; [Tyr306*]	p.[Leu642Pro]; [Leu642Pro]	p.[Val708Alafs*5]; [Val708Alafs*5]	p.[Arg632*]; [Arg632*]	p.[Pro274Leufs*18]; [Trp750Arg]	p.[Glu338*]; [Arg644*]	p.[Glu766*]; [Glu795Glyfs*3]	p.[Ala975Profs*22]; [Ala975Profs*22]
Gestational Age (weeks); Birth parameters	NA	NA	NA	NA	34 w; NA	NA	NA	NA	30 weeks. W: 1.2 kg, L: 44 cm	36 weeks. W: 2.4kg, L 47 cm	34 weeks. NA	Term. NA	35 week. W: 2kg	NA	32 weeks. NA
Height (cm)	NA	NA	NA	NA	192.6	164	178	191.5	150 (target: 157)	174.5 cm	NA	NA	NA	NA	140
Weight (kg)	NA	NA	NA	NA	87	NA	53	67	52	80	NA	BMI22	NA	NA	35
Head circumference (cm)	NA	NA	NA	NA	56	NA	53	56	NA	NA	NA	NA	NA	NA	50.5
Craniofacial features															
Alopecia/Thin sparse hair	NA	NA	-	-	+	+	+	NA	+	-	NA	+	+	+	+
Atypical Dentition	Abnormal alignment	Abnormal alignment	Retains a single baby tooth	NA	Severe caries	Frequent caries	NA	NA	Pyorrhea, complete dental loss at age 14	Multiple caries, periodontal disease	NA	Abnormal alignment	NA	Multiple caries	-
Narrow thin facies/ narrow high palate	+	+	-	-	-	-	NA	NA	+	-	-	+		+	+
Webbed neck	+	+	-	-	+	-	NA	NA	-	+	NA	-	-	-	-

NA: features not reported; POTS: postural orthostatic tachycardia syndrome; SMA: Superior mesenteric artery; GER: Gastroesophageal reflux; †Prematurely aged appearance included ptosis, and sagging cheeks. , ‡, §, ¶

Family No.	I		II	III	IV	V	VI		VII	VIII	IX	X	XI	XII	XIII
Other craniofacial features	Low posterior hairline, large ears		-	Micrognathia	Asymmetrical face, hypertelorism, low-set and posteriorly rotated ears with attached earlobes	Mild ptosis	NA	NA	Elongated uvula	Cleft palate, down-slanting palpebral fissures, epicanthus, deep set eyes, malar hypoplasia, low set ears, micro/retrognathia	NA	Bilateral notched earlobes	Bilateral notched earlobes	Narrow nose	Thin nasal root
**Cutaneous Features**															
Skin hyperextensibility	+	+	+	+	+	+	+	+	+	+	+	+	+	+	+
Thin, translucent skin	NA	NA	NA	+	+	+	+	+	-	+	+	+	-	+	+
Excessive skin/ skin folding	+	+	+	+	+	-	+	+	+	+	+	+	+	+	+
Atrophic scars/ Delayed wound healing	+	+	+	+	+	+	+	+	+	+	+	+	+	+	+
Easy bruising	+	NA	+	+	+	+	+	+	+	+	+	+	+	+	+
Piezogenic papules	NA	NA	+	+	NA	NA	NA	NA	+	+	NA	NA	+	-	-
Prematurely aged appearance	+	+	No; acrogeria only	NA	+	NA	+	+	+	No, acrogeria only	NA	NA	NA	No, acrogeria only	+
**Skeletal Features**															
Generalized joint hypermobility (Beighton score)	+ (8/9)	+ (NA)	+ (8/9)	+ (8/9)	+ (NA)	+ (9/9)	+ (6/9)	- (2/9)	+ (5/9)	+ (7/9)	+ (NA)	+ (3/9)	+ (9/9)	+ (8/9)	+ (9/9)
Congenital hip dislocation	-	-	-	+	-	-	-	NA	NA	NA	-	+	-	+	+
Other Dislocations; Subluxations	+; NA	+; NA	+; +	+; NA	+; +	NA; +	+; +	+; NA	+; NA	NA; +	-	+; NA	+; NA	+	-; -
Pes planus	+	+	+	+	+	+	+	+	+	-	+	+	+	+	+
Spine deformities	-	NA	-	+	-	+	+	+	+	+	-	+	-	-	+
Hallux valgus	+	+	+	+	+	-	-	-	+	-	NA	+	-	-	-
Hammertoes	+	+	+	+	+	-	-	-	-	+	NA	+	-	-	-
Osteopenia	+	+	+	+	NA	-	NA	NA	+	-	+	-	+	NA	-

NA: features not reported; POTS: postural orthostatic tachycardia syndrome; SMA: Superior mesenteric artery; GER: Gastroesophageal reflux; †Prematurely aged appearance included ptosis, and sagging cheeks. , ‡, §, ¶

Family No.	I		II	III	IV	V	VI		VII	VIII	IX	X	XI	XII	XIII
Other skeletal features	NA	NA	NA	Down slanting shoulders, severe hip osteoarth-ritis	Pectus excav-atum, multiple ankle distortion	Down slanting shoulders, hip dysplasia, ankle sprains	Arachno dactyly, wrist and thumb signs, systemic score 8	Pectus excav-atum, aracnoi-dactyly, wrist sign, systemic score 7, hindfoot deform-ity	Rotator cuff disease & impingem ent, epithroch-leitis, gonar-throsis, patellar instability, Achilles, tendon-opathy,	Short stubby fingers, hips, dysmetria, absence and hypoplasia of toes	NA	Narrow-ing at C4-5 and C6-7, severe degenera -tive change to L2 to L5	Bilat-eral talipes equino varus, osteo-arth-ritis	Toe/ elbow joint deform -ity, ankle instabil ity and sprains	Patella instabil-ity
Neuromuscular features															
Neonatal hypotonia	+	-	-	NA	NA	NA	NA	NA	+	+	-	NA	NA	-	-
Delayed motor development	-	-	+	NA	NA	NA	NA	NA	+	+	+	NA	NA	-	-
Muscle hypotrophy/ hypoplasia	NA	+	NA	NA	NA	+, progressive	NA	NA	Scapular girdle	Pectorali major, diastasis recti	NA	NA	NA	-	-
Cardiovascular features															
Mitral prolapse/ regurgitation	-	-	Pro-lapse	Regurg--itation	Prolapse	-	Prolapse	-	-	Regurg--itation	NA	-	-	-	Pending echocardi ogram
Aortic root dilation	-	-	NA	+	-	-	-	-	NA	-	-	+	NA	NA	
Hematoma	-	-	NA	NA	NA	+	-	-	NA	+	-	+	+	NA	-
Varicose veins	-	-	NA	NA	NA		-	+	+	+	-		-	NA	+
Other cardiovascular features	-	-	NA	Bilateral stenosis of carotids	NA	POTS	Circular peri-cardial effusion	-	Peripheral artery disease	Regurg-itation at tricuspid, pulmonary vales	SMA rupture	Dissec-tion, dilation, aberrant, tortuo-sity	Tran-sient isch-aemic attack, atrial flutter	SMA aneur-ysm and ruptur e	-
Other organ systems															
Gastrointestinal features	NA	NA	Chronic constip -ation	Bowel rupture	-	GER, dysphagia, bloating, cramps, constip-ation, diarrhea	NA	NA	-	NA	Bowel rupture, paralytic ileus	NA	-	-	-, small appetite
Hernias	Umbilical/ ventral/ inguinal hernias	NA	-	Surgical site	Hernia-tion of fat in the right armpit	NA	+	-	Umbilical hernia	Inguinal hernia	Surgical site	NA	-	Spinal disc hernia, umbilic al hernia	Spinal disc hernia

NA: features not reported; POTS: postural orthostatic tachycardia syndrome; SMA: Superior mesenteric artery; GER: Gastroesophageal reflux; †Prematurely aged appearance included ptosis, and sagging cheeks. , ‡, §, ¶

Family No.	I		II	III	IV	V	VI		VII	VIII	IX	X	XI	XII	XIII
Urogenital features	NA	NA	Cryptor-chid-ism	NA	NA	Bladder cramps, urinary retention/ urgency	NA	Cryptor---chidism	NA	Cryptorchid ism	Ovarian mucinous cystaden-oma, urinary retention/ urgency	NA	-	-	-
Other features	Diabetes mellitus, cellulitis	NA	Im-paired temper-ature sensa-tion, kerato-conjun-ctivitis, sicca	Elbow bursitis, hypertri-glycerid-emia	Spontan-eous pneumo-thorax, myopia, tinnitus	Chronic fatigue, chronic pain, myopia,	NA	Strabis-mus, astigma--tism, myopia	Vocal cord nodules, subcut-aneous spheroids, multiple papules with follicular promin-ence, chronic fatigue, myopia, astigma-tism	Chronic pain	NA	Chronic fatigue	High blood pres-sure, hypo-thyroid-ism, pre-dia-betes, visual loss in right eye, small old left temp-oral lacunar infarct	Myopia	Chronic fatigue, pain

NA: features not reported; POTS: postural orthostatic tachycardia syndrome; SMA: Superior mesenteric artery; GER: Gastroesophageal reflux; †Prematurely aged appearance included ptosis, and sagging cheeks. , ‡, §, ¶
